# Analgesic effect of ultrasound-guided transversus abdominis plane block with or without rectus sheath block in laparoscopic cholecystectomy: a randomized, controlled trial

**DOI:** 10.1186/s12871-024-02590-x

**Published:** 2024-06-08

**Authors:** Jung-Pil Yoon, Hee Young Kim, Jieun Jung, Jimin Lee, Seyeon Park, Gyeong-Jo Byeon

**Affiliations:** 1 Department of Anesthesia and Pain Medicine, Pusan National University Yangsan Hospital, Pusan National University School of Medicine, Geumoro 20, Yangsan, Gyeongnam 50612 Republic of Korea; 2 Research Institute for Convergence of Biomedical Science and Technology, Pusan National University Yangsan Hospital, Yangsan, Republic of Korea

**Keywords:** Block, ERAS, Laparoscopic cholecystectomy, Rectus sheath, Sleep quality, Transversus Abdominis plane block

## Abstract

**Background:**

Ultrasound-guided transversus abdominis plane (TAP) block is commonly used for pain control in laparoscopic cholecystectomy. However, significant pain persists, affecting patient recovery and sleep quality on the day of surgery. We compared the analgesic effect of ultrasound-guided TAP block with or without rectus sheath (RS) block in patients undergoing laparoscopic cholecystectomy using the visual analog scale (VAS) scores.

**Methods:**

The study was registered before patient enrollment at the Clinical Research Information Service (registration number: KCT0006468, 19/08/2021). 88 American Society of Anesthesiologist physical status I-III patients undergoing laparoscopic cholecystectomy were divided into two groups. RS-TAP group received right lateral and right subcostal TAP block, and RS block with 0.2% ropivacaine (30 mL); Bi-TAP group received bilateral and right subcostal TAP block with same amount of ropivacaine. The primary outcome was visual analogue scale (VAS) for 48 h postoperatively. Secondary outcomes included the use of rescue analgesics, cumulative intravenous patient-controlled analgesia (IV-PCA) consumption, patient satisfaction, sleep quality, and incidence of adverse events.

**Results:**

There was no significant difference in VAS score between two groups for 48 h postoperatively. We found no difference between the groups in any of the secondary outcomes: the use of rescue analgesics, consumption of IV-PCA, patient satisfaction with postoperative pain control, sleep quality, and the incidence of postoperative adverse events.

**Conclusion:**

Both RS-TAP and Bi-TAP blocks provided clinically acceptable pain control in patients undergoing laparoscopic cholecystectomy, although there was no significant difference between two combination blocks in postoperative analgesia or sleep quality.

## Introduction

Laparoscopic cholecystectomy causes less postoperative pain than open cholecystectomy, but it still causes significant pain. Pain after laparoscopic cholecystectomy is related to multi-factors such as incisional, visceral, and referred shoulder pain. It is known that incisional pain is more dominant than visceral pain on the day of laparoscopic cholecystectomy [1]. Postoperative pain is one of the primary reasons for prolonged hospitalization and delayed recovery after surgery [2]. In addition, postoperative pain is a major cause of sleep disturbance after surgery [3], which can also lead to delayed recovery, increased morbidity, higher risk of delirium, and increased occurrence of cardiovascular events [4]. Therefore, multi-modal analgesia is widely accepted as an important component for enhanced recovery after laparoscopic surgery [5].

The ultrasound-guided transversus abdominis plane (TAP) block is one of the preferred methods of analgesia for surgery of the anterolateral abdominal wall and has been extensively used for pain control in laparoscopic cholecystectomy [6]. Nonetheless, there are discrepancies in outcomes; uncertainty persists regarding different TAP strategies for laparoscopic cholecystectomy [5], and midline pain is yet to be addressed [7–9].

Laparoscopic cholecystectomy typically involves three or four incisions: one umbilical, one subxiphoid, and one or two subcostal ports [10]. An umbilical incision into which a laparoscope is usually inserted with a large-bore trocar induces the most intense pain, which is dominant in the first 48 h after laparoscopic cholecystectomy [11]. In a variety of abdominal surgeries, the rectus sheath (RS) block has been reported to be effective in reducing postoperative pain from midline incisions, including the umbilical or periumbilical areas [7, 12, 13]. Consequently, we hypothesized that the addition of RS block to TAP block may be beneficial for pain management in laparoscopic cholecystectomy in comparison to TAP block alone by providing coverage for pain in the midline incision. However, few studies have evaluated the analgesic effect of the RS block compared to the TAP block in laparoscopic cholecystectomy.

In this study, we evaluated the analgesic effect of the RS block in addition to the TAP block compared to the TAP block alone on postoperative pain management in laparoscopic cholecystectomy.

## Methods

### Patients

This prospective, randomized, controlled study was approved by the Institutional Review Board of Pusan National University Yangsan Hospital (approval number: 05–2021 − 155, 21/07/2021); written informed consent was obtained from all patients in the study. The study was registered before patient enrollment at the Clinical Research Information Service (registration number: KCT0006468, 19/08/2021) and conducted in accordance with the principles of the Declaration of Helsinki. A total of 88 patients with American Society of Anesthesiology (ASA) physical status I-III, age between 19 and 80 years, who were scheduled for laparoscopic cholecystectomy were enrolled in this study. The exclusion criteria were as follows: history of coagulation disorders including coagulation factor deficiency, thrombocytopenia, platelet dysfunction, allergic reactions to ropivacaine, neurological defects in the procedural area, pregnancy, lack of understanding of the study, or inability to respond appropriately to the questionnaires. The patients were randomly allocated to one of two groups using a computer-generated randomized sequence table with an allocation ratio of 1:1 and a block size of two. The study was conducted using a sealed envelope system. The sealed, opaque, sequentially numbered envelopes were opened by intervention staff who conducted induction of anesthesia and block procedure just prior to surgery. A second investigator involved in the assessment of postoperative outcomes and another investigator involved in data collection were blinded to group allocation.

### Anesthesia management

Standard anesthesia monitoring recommended by the ASA was continuously conducted, and general anesthesia was induced using 1–2 mg/kg of 1% propofol and 0.5-1.0 mcg/kg/min of remifentanil. After loss of consciousness, 0.8 mg/kg of rocuronium was administered, and endotracheal intubation was performed. To maintain anesthesia, the end-tidal sevoflurane concentration was adjusted between 2 and 4 vol% with oxygen (50%) and air (50%). The bispectral index score was maintained between 40 and 60 for the depth of anesthesia, and the end-tidal carbon dioxide partial pressure was maintained within the range of 35–40 mmHg. For intraoperative pain control, remifentanil (2–5ng/mL of effect-site concentration) was continuously infused using a target-controlled infusion pump to maintain systolic blood pressure within 20% of baseline values. At the end of the surgery, all patients received 0.3 mg of ramosetron for the management of postoperative nausea and vomiting (PONV). For postoperative pain management, all patients received an intravenous patient-controlled analgesic (IV-PCA) pump (Anaplus®; Ewha Biomedics, Seoul, Korea). For IV-PCA according to the non-opioid regimen in our hospital, 120 mg ketorolac, 80 mg nefopam, and 0.3 mg ramosetron were mixed with saline to a total volume of 60 mL. The basal infusion rate was 1 mL/h, the volume of patient-required bolus was 1 mL, and the lock-out time was 15 min.

### Block procedure

**Fig. 1:**
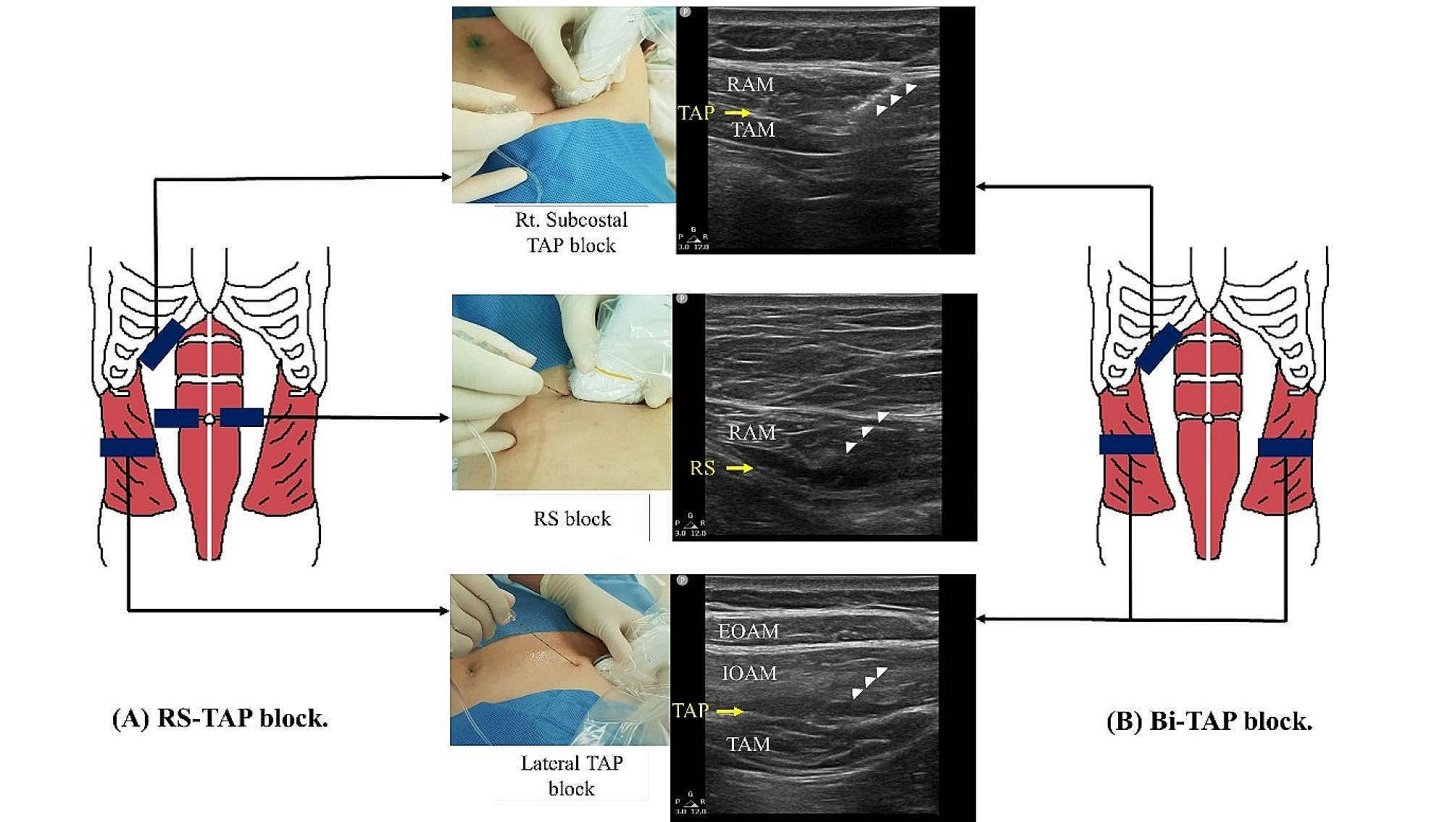
Illustration of US-guided TAP and RS blocks. (A) RS-TAP block consists of right subcostal TAP, right lateral TAP, and bilateral RS block. (B) Bi-TAP block consists of right subcostal TAP and bilateral TAP block. The white arrow head indicates block needle. Rt., right; Lt., left; RS, rectus sheath; RAM, rectus abdominis muscle; TAP, transversus abdominis plane; TAM, transversus abdominis muscle; EOAM, external oblique abdominis muscle; IOAM, internal oblique abdominis muscle; US, ultrasound

After the induction of anesthesia and with stable vital signs, block procedures were performed by investigators skilled in ultrasound-guided blocks. For block procedure, a portable ultrasound (CX 50, Phillips, Eindhoven, Netherlands) was used with a high-frequency (5.0–13.0 MHz) linear probe transducer. In this study, the RS-TAP block was defined as a combination of right subcostal, right unilateral TAP, and bilateral RS blocks. The Bi-TAP block was composed of right subcostal and bilateral TAP blocks (Fig. 1). In both groups, the total amount of local anesthetic was 30 mL of ropivacaine 0.2%. In the RS-TAP group, the blocks were performed in the following order: right subcostal TAP, right lateral TAP, and bilateral RS block. For the right subcostal TAP block, the probe below the xiphoid process was advanced towards the right lateral along the subcostal margin, keeping the rectus abdominis and transversus abdominis muscles in view. A block needle was then inserted from the medial-to-lateral approach through an in-plane technique, until the needle tip reached the fascial plane between the posterior RS and anterior margin of the transversus abdominis muscle; 10 mL of 0.2% ropivacaine was then spread out on the fascial plane medial to the linea semilunaris. Accordingly, a right lateral TAP block was performed. The probe was placed on the mid-axillary line between the subcostal margin and iliac crest, and 10 mL of 0.2% ropivacaine was administered to the right TAP. For the RS block, the linea alba was scanned above the umbilicus and the probe was moved laterally to identify the rectus abdominis muscle. A block needle was inserted until the needle tip reached on the posterior RS of the rectus abdominis muscle, with the transversalis fascia of the peritoneum underneath. Bilateral RS block were performed using 5 mL ropivacaine 0.2% on each side. In the Bi-TAP group, the block was performed at the three points: right subcostal; and bilateral TAP. 10 mL of 0.2% ropivacaine was injected into the right subcostal and bilateral TAP. Therefore, the actual difference in the block procedure between two groups was that bilateral RS block in the RS-TAP group and left TAP block in the Bi-TAP group. All patients in both groups received right subcostal and right lateral TAP block. (Fig. 1).

### Outcome measurements

Postoperative pain intensity was the primary outcome measured using the visual analog scale (VAS) scores at the post-anesthesia care unit (PACU) and at 12, 24, and 48 h postoperatively, with scores ranging from 0 points for no pain to 100 points for unbearable pain. For postoperative pain control, infusion of IV-PCA was initiated in the PACU, and the cumulative total consumption of IV-PCA was recorded 24 and 48 h after surgery. When the VAS score was > 60 and the patient wanted additional analgesics, 90 mg of diclofenac was administered intramuscularly. If pain control was unsatisfactory with the first analgesic, 25 mg of meperidine was injected intravenously with precaution of narrow safety margin. The number of patients who required rescue analgesics was also recorded. Patients requiring more than one analgesic were counted only once. In addition, patient satisfaction with pain control was evaluated 48 h after surgery using a 5-point Likert scale (5 = very satisfied, 4 = satisfied, 3 = neutral, 2 = dissatisfied, and 1 = very dissatisfied). Sleep quality on the night of surgery was assessed on the day after surgery using a 3-item questionnaire: total hours of sleep, number of awakenings during the night, and reasons for sleep disturbance. Satisfaction with sleep quality was also assessed using a 5-point Likert scale (5 = very satisfied, 4 = satisfied, 3 = neutral, 2 = dissatisfied, and 1 = very dissatisfied). The incidence of PONV, dizziness, hypotension, persistent paresthesia at the block site, and urinary retention was recorded 48 h after surgery.

### Sample size estimation

This study compared the differences in postoperative pain intensity between RS-TAP and Bi-TAP groups of patients undergoing laparoscopic cholecystectomy. We considered a clinically significant difference when the mean difference (${\mu }_{c}-{\mu }_{t}$) in VAS measured at 12 h after surgery was 10 or more. In the previous study [7], the result of VAS measured 12 h after surgery in the Bi-TAP group was 30.59 ± 15.94, the measured standard deviation (σ) was 15.94. The calculated sample size for this study was 44 patients per group when we considered type I error (α) = 0.05, type II error (β) = 0.2, and a predicted dropout rate of 10%.

$$n =\frac{2{\left({z}_{\alpha /2}+{z}_{\beta }\right)}^{2}{\sigma }^{2}}{\left({\mu }_{c}-{\mu }_{t}\right)}$$

$${z}_{\alpha /2}$$= 1.95996, $${z}_{\beta }$$= 0.84162, σ = 15.94, $${\mu }_{c}-{\mu }_{t}$$= 10

### Statistical methods

For the statistical analysis of all measurements, IBM SPSS Statistics for Windows (version 27.0; IBM Corp., Armonk, NY, USA) was used. Numerical or categorical data are reported as mean ± standard deviation or number of patients (%), respectively. The Kolmogorov–Smirnov test was used to check the normality of the numerical data. Student’s *t*-test was used to compare the means of normally distributed numerical data for age, height, weight, anesthesia time, VAS score, consumption of IV-PCA, sleep time, and number of awakenings between the two study groups. For comparison of categorical data, such as ASA physical status, sex, number of rescue analgesics administered, reason to wake up, satisfaction with sleep quality and postoperative pain control, and adverse events, the chi-squared or Fisher’s exact test was used. Statistical significance was set at *p* < 0.05, and was considered statistically significant.

## Results

### Patient characteristics

**Fig. 2:**
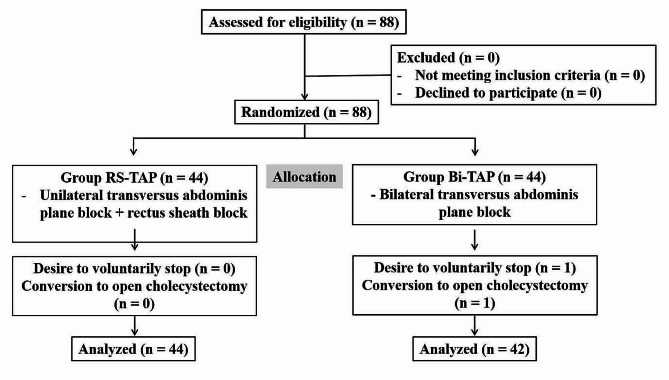
Patient enrollment and a study flowchart. RS-TAP, rectus sheath- transversus abdominis plane; Bi-TAP, bilateral transversus abdominis plane

Of the 88 patients assessed for eligibility, none were excluded, and patients were divided into two groups of 44 each. In the RS-TAP group, 44 patients were studied with no dropouts. Two patients dropped out in the Bi-TAP group; one of them refused to continue with the study when the investigator (B) went to the ward to check the VAS score the day after surgery, while surgery for the other patient was converted from laparoscopic cholecystectomy to open cholecystectomy. Therefore, this patient was excluded from the study after being explained the reason for dropping out (Fig. 2). The demographic and preoperative characteristics were not significantly different between the two groups (Table 1).

**Table 1:** Demographic data

Characteristics	RS-TAP (*n* = 44)	Bi-TAP (*n* = 42)	*P*-value
ASA PS (I/II/III)	19/24/1	23/20/1	0.689
Sex (M/F)	19/25	23/21	0.522
Age (years)	57.6 ± 13.0	59.1 ± 10.0	0.504
Height (cm)	162.2 ± 10.8	160.5 ± 8.1	0.089
Weight (kg)	66.0 ± 14.0	67.2 ± 8.7	0.126
Anesthesia time (hours)	1.6 ± 0.4	1.7 ± 0.3	0.483

All measured values are presented as mean ± standard deviation or number of patients. ASA PS, American Society of Anesthesiologist physical status; RS-TAP, rectus sheath-transversus abdominis plane; Bi-TAP, bilateral transversus abdominis plane

### Primary and secondary outcomes

Both groups had the highest VAS scores at 12 h postoperatively and the lowest at 48 h postoperatively, but there was no significant intergroup differences in VAS scores at any time point during postoperative day 2 (12 h: RS-TAP group 55.7 ± 24.0 vs. Bi-TAP group 58.5 ± 26.9, *P* = 0.615; 48 h: RS-TAP group 24.1 ± 9.7 vs. Bi-TAP group 26.8 ± 12.5, *P* = 0.266) (Table 2).

**Table 2:** The VAS, number of patients requiring rescue analgesics, consumption of IV-PCA, and patient satisfaction with postoperative pain control after surgery

Pain control	Time	RS-TAP (*n* = 44)	Bi-TAP (*n* = 42)	*P*-value
**VAS**	0 h	34.1 ± 20.8	31.2 ± 18.6	0.498
	12 h	55.7 ± 24.0	58.5 ± 26.9	0.615
	24 h	33.9 ± 14.5	35.8 ± 19.7	0.598
	48 h	24.1 ± 9.7	26.8 ± 12.5	0.266
**Number of patients requiring rescue analgesics**	0 h	11 (25.0)	15 (35.7)	0.350
	12 h	12 (27.3)	13 (31.0)	0.813
	24 h	3 (6.8)	4 (9.5)	0.710
	48 h	2 (4.5)	4 (9.5)	0.428
**Consumption of IV-PCA (mL)**	24 h	35.2 ± 10.9	36.3 ± 10.1	0.650
	48 h	54.9 ± 10.6	56.6 ± 7.0	0.388
**Patient satisfaction**	48 h			0.241
5 = very satisfied		4 (9.1)	8 (19.0)	
4 = satisfied		21 (47.7)	14 (33.3)	
3 = neutral		14 (31.8)	11 (26.2)	
2 = dissatisfied		5 (11.4)	9 (21.4)	
1 = very dissatisfied		0 (0.0)	0 (0.0)	

All measured values are presented as mean ± standard deviation or number of patients (%). Zero h, when the patient was in the post-anesthetic care unit (PACU); VAS, visual analog scale; IV-PCA, intravenous patient-controlled analgesia; RS-TAP, rectus sheath- transversus abdominis plane; Bi-TAP, bilateral transversus abdominis plane

The number of patients receiving rescue analgesics when the VAS score was 60 was not significantly different between groups. In addition, there were no significant difference between the two groups in the consumption of IV-PCA measured at 24 and 48 h after surgery or patient satisfaction with postoperative pain control (Table 2).

There were no differences between the groups in any component of sleep quality; the total hours of sleep and number of awakenings on the night of surgery did not differ between the two groups. There was also no significant difference in the reasons for awakening between the two groups. The most common reason for sleep disturbance was noise at night, followed by unfamiliar environment, pain, and toilet use. There was no significant difference in sleep satisfaction between the two groups (Table 3).

**Table 3:** Postoperative sleep quality among patients

Sleep quality		RS-TAP (*n* = 44)	Bi-TAP (*n* = 42)	*P* value
Sleep time (hours)		6.1 ± 2.1	5.8 ± 2.5	0.543
Number of awakenings (times)		3.5 ± 2.7	4.0 ± 3.0	0.343
The reasons for sleep disturbance	Using toilet	7 (15.9)	6 (14.3)	1.000
	Noise	18 (40.9)	14 (33.3)	0.509
	Foreign environment	13 (29.5)	13 (31.0)	1.000
	Pain	8 (18.2)	12 (28.6)	0.312
Satisfaction	5 = very satisfied	10 (22.7)	8 (19.0)	0.824
	4 = satisfied	14 (31.8)	10 (23.8)	
	3 = neutral	10 (22.7)	10 (23.8)	
	2 = dissatisfied	7 (15.9)	10 (23.8)	
	1 = very dissatisfied	3 (6.8)	4 (9.5)	

All measured values are presented as mean ± standard deviation or number of patients (%). RS-TAP, rectus sheath transversus abdominis plane; Bi-TAP, bilateral transversus abdominis plane

The incidence of adverse events was not significantly different between the two groups. The most common adverse event was PONV (29.5% and 11.9% in the RS-TAP and Bi-TAP groups, respectively), followed by dizziness, urinary retention, and paresthesia at the needle insertion site (Table 4).

**Table 4:** Adverse events

Adverse events	RS-TAP (*n* = 44)	Bi-TAP (*n* = 42)	*P*-value
Postoperative nausea and vomiting	13 (29.5)	5 (11.9)	0.063
Dizziness	5 (11.4)	3 (7.1)	0.714
Paresthesia	0 (0.0)	1 (2.4)	0.488
Urinary retention	1 (2.3)	2 (4.8)	0.612

All measured values are presented as the number of patients (%). RS-TAP, rectus sheath transversus abdominis plane; Bi-TAP, bilateral transversus abdominis plane

## Discussion

In this study, we evaluated the analgesic effect of two different combination blocks for postoperative pain control in laparoscopic cholecystectomy: one for right subcostal, right unilateral TAP and RS block, and the other for right subcostal and bilateral TAP block. The addition of an RS block to a right subcostal and right lateral TAP block did not further improve pain scores compared to right subcostal and bilateral TAP block in patients undergoing laparoscopic cholecystectomy. There were no significant differences in the use of rescue analgesics, the use of IV-PCA, patient-reported satisfaction with pain management, or sleep quality between the two groups.

A few studies have reported the effectiveness of a combination block with RS and TAP blocks in upper abdominal surgeries. However, previous studies compared the analgesic effect of combining RS and TAP blocks with wound site infiltration [12, 14]. In a pilot study comparing two different block procedures between the combination of RS and oblique subcostal TAP blocks and oblique subcostal block alone in laparoscopic cholecystectomy, there was no difference in postoperative VAS scores and patient satisfaction with pain management [15]. Similarly, this study found no significant difference in postoperative analgesia between the RS-TAP and Bi-TAP groups. This may be due to sufficient analgesia with the right subcostal TAP block performed in both groups. In our hospital, we performed right subcostal TAP block in all patients undergoing laparoscopic cholecystectomy for effective pain control based on substantial evidence that the subcostal TAP block provides excellent postoperative analgesia for laparoscopic cholecystectomy [14, 16–19], contrary to the inconsistent results of the bilateral TAP block [7–9, 16, 17]. Therefore, the subcostal TAP block may have obscured the beneficial effects of RS block. However, a recent study showed that the subcostal TAP block resulted in a heterogeneous cutaneous sensory block area with variable distribution, mostly covering the upper medial part of the abdomen [20]. Consequently, the analgesic effect of subcostal TAP block on the periumbilical area is still controversial.

Another key factor affecting the efficacy of RS block is the optimal dose required for postoperative pain control, and there is no standardized consensus on the clinically effective and safe dose for an abdominal fascial plane block. In general, TAP and RS blocks require large volumes of local anesthetic to spread around multiple segmental thoracoabdominal nerves within the fascial plane. A previous systematic review on the efficacy of the TAP block during laparoscopic cholecystectomy found that at least 20 ml of local anesthetic was required for the TAP block [6]. Meanwhile, local anesthetics (LA) carry the potential risk of local anesthetic systemic toxicity (LAST). Although the incidence of LAST in RS or TAP block is as low as 0-0.8% [21, 22], it is a fatal complication. The symptoms vary from mild central nervous system disturbances to cardiac instability, leading to cardiovascular collapse and death in a dose-dependent manner [23]. To ensure both safety and efficacy, and to use the same amount of LA in both groups, we administered 10 mL of 0.2% ropivacaine to each TAP block, and 5 mL of 0.2% ropivacaine to each side of the rectus abdominis muscle for the RS block, so that the total dose of ropivacaine was 60 mg for each group. This was within the safe range of LAST, which was lower than 150 mg of ropivacaine, suggested as an acceptable threshold for acute central nervous system toxicity in the literature [22, 24]. Nonetheless, the dose may not have been sufficient to provide effective analgesia, and this may have influenced the results of our study. Although not included in the present study, the addition of local anesthetic adjuvants in TAP block, such as dexmedetomidine or epinephrine, may have allowed for the use of a sufficient volume of LA while avoiding the risk of LAST by lowering the peak plasma concentration of LA [25, 26]. Therefore, further research is needed to investigate the effective analgesic dose of LA at a safe threshold for a combination block.

To the best of our knowledge, this is the first trial to evaluate the effect of the RS-TAP block on postoperative pain control in terms of patients’ self-reported sleep disturbance together with objective pain scores after laparoscopic cholecystectomy. Generally, there is a reciprocal relationship between postoperative sleep disturbance and pain; sleep disturbance increases pain sensitivity and may contribute to pain exacerbation, whereas pain and opioids disrupt sleep quality by decreasing rapid eye movement (REM), stimulating frequent sleep arousal, and sleep fragmentation [3, 27]. However, we did not find any differences between the groups in terms of any of the components of sleep quality, including total sleep time or number of awakenings. Multiple factors, such as surgical inflammatory response, severity of surgical trauma, pain, anxiety, noise, and light affect postoperative nocturnal sleep [3], but the presence of pain and environmental factors are known to be the predominant factors responsible for sleep disturbance at night of the first postoperative day [28]. Similar to previous studies, the most common reason for waking at night in both groups in this study was noise, followed by environmental changes. However, pain affected only 18.2% and 28.6% of the patients with RS-TAP and Bi-TAP blocks, respectively, which may indicate that pain was adequately controlled by either block. Sleep quality has been relatively neglected, but is an important component in the context of postoperative recovery or patient satisfaction with pain control in modern anesthesia [29]. Thus, further studies are required to minimize sleep disturbance as well as to improve pain management.

Our study had several limitations. First, we could not accurately assess the true extent of the block because it was administered after the induction of anesthesia. To assess the actual extent of the blocked sensory nerves in the abdominal fascial plane, the block should be administered before induction of anesthesia. However, many previous trials have also been performed after the induction of anesthesia, as an abdominal fascial plane block is a safe procedure with a reported technical complication rate of 2.4% [21]. Second, pain characteristics were not analyzed in this study, and whether the most severe postoperative pain was somatic or visceral could not be determined. This may have been helpful in assessing the effectiveness of somatic pain control using the RS-TAP block or the need for further visceral pain control. Third, we did not record the duration of pneumoperitoneum, so could not evaluate the effect of residual pneumoperitoneum on postoperative pain intensity after laparoscopic cholecystectomy. However, at the end of surgery the residual gas was actively aspirated by suction and removed from the peritoneal cavity as much as possible. Therefore, we expect that the duration of pneumoperitoneum may not have much effect on postoperative pain severity. Fourth, evaluation of the sleep quality was done based on the results obtained through a patient self-report survey, which relied on the patients’ subjective recall; this may have introduced patient recall bias. In addition, we did not survey preoperative baseline sleep patterns; therefore, we could not determine the level of deterioration in sleep quality as compared to that prior to surgery. Therefore, further high-quality research is needed using a more structured questionnaire or a clinically applicable objective sleep assessment tool such as polysomnography to assess perioperative sleep quality.

Despite these limitations, our study has several significant clinical implications. We found that the combination of RS and unilateral TAP block was as effective as the bilateral TAP block for postoperative pain control in patients undergoing laparoscopic cholecystectomy. Although it is cautious to recommend one over the other based on our research, the RS block is an easy, simple, and safe block and could be considered as part of a multidisciplinary, multi-modal analgesia for enhanced recovery after surgery (ERAS) in laparoscopic cholecystectomy.

In conclusion, there was no significant difference between two combination blocks of Bi-TAP and RS-TAP in reducing postoperative pain management and sleep quality after laparoscopic cholecystectomy. However, both the RS-TAP and Bi-TAP blocks provided a clinically acceptable level of effective pain control in patients undergoing laparoscopic cholecystectomy.

## Data Availability

The datasets used and analyzed in the current study are available from the corresponding author on reasonable request.

## References

[1] Bisgaard T, Klarskov B, Rosenberg J, Kehlet H (2001). Characteristics and prediction of early pain after laparoscopic cholecystectomy. Pain.

[2] Gan TJ (2017). Poorly controlled postoperative pain: prevalence, consequences, and prevention. J Pain Res.

[3] Rampes S, Ma K, Divecha YA, Alam A, Ma D (2019). Postoperative sleep disorders and their potential impacts on surgical outcomes. J Biomed Res.

[4] Su X, Wang DX (2018). Improve postoperative sleep: what can we do?. Curr Opin Anaesthesiol.

[5] Barazanchi AWH, MacFater WS, Rahiri JL, Tutone S, Hill AG, Joshi GP. collaboration P. Evidence-based management of pain after laparoscopic cholecystectomy: a PROSPECT review update. Br J Anaesth. 2018;121(4):787–803. 10.1016/j.bja.2018.06.023. 10.1016/j.bja.2018.06.023 30236241

[6] Alsharari AF, Abuadas FH, Alnassrallah YS, Salihu D. Transversus Abdominis Plane Block as a strategy for effective Pain Management in patients with Pain during laparoscopic cholecystectomy: a systematic review. J Clin Med. 2022;11(23). 10.3390/jcm11236896. 10.3390/jcm11236896 PMC9735918 36498471

[7] Choi YM, Byeon GJ, Park SJ, Ok YM, Shin SW, Yang K (2017). Postoperative analgesic efficacy of single-shot and continuous transversus abdominis plane block after laparoscopic cholecystectomy: a randomized controlled clinical trial. J Clin Anesth.

[8] El-Dawlatly AA, Turkistani A, Kettner SC, Machata AM, Delvi MB, Thallaj A, Kapral S, Marhofer P (2009). Ultrasound-guided transversus abdominis plane block: description of a new technique and comparison with conventional systemic analgesia during laparoscopic cholecystectomy. Br J Anaesth.

[9] Petersen PL, Stjernholm P, Kristiansen VB, Torup H, Hansen EG, Mitchell AU, Moeller A, Rosenberg J, Dahl JB, Mathiesen O (2012). The beneficial effect of transversus abdominis plane block after laparoscopic cholecystectomy in day-case surgery: a randomized clinical trial. Anesth Analg.

[10] Casaccia M, Palombo D, Razzore A, Firpo E, Gallo F, Fornaro R. Laparoscopic single-Port Versus Traditional Multi-port Laparoscopic Cholecystectomy. JSLS. 2019;23(3). 10.4293/JSLS.2018.00102. 10.4293/JSLS.2018.00102 PMC6708410 31488940

[11] Siddiqui NA, Azami R, Murtaza G, Nasim S (2012). Postoperative port-site pain after gall bladder retrieval from epigastric vs. umbilical port in laparoscopic cholecystectomy: a randomized controlled trial. Int J Surg.

[12] Abdelsalam K, Mohamdin OW (2016). Ultrasound-guided rectus sheath and transversus abdominis plane blocks for perioperative analgesia in upper abdominal surgery: a randomized controlled study. Saudi J Anaesth.

[13] Hong S, Kim H, Park J (2019). Analgesic effectiveness of rectus sheath block during open gastrectomy: a prospective double-blinded randomized controlled clinical trial. Med (Baltim).

[14] Tor IH, Celik EC, Aydin ME (2020). Analgesic effect of combined transversus abdominis plane block and rectus sheath block in laparoscopic cholecystectomy: prospective randomized study. Ain Shams J Anesthesiology.

[15] Ramkiran S, Jacob M, Honwad M, Vivekanand D, Krishnakumar M, Patrikar S (2018). Ultrasound-guided combined Fascial Plane blocks as an intervention for Pain Management after laparoscopic cholecystectomy: a Randomized Control Study. Anesth Essays Res.

[16] Bhatia N, Arora S, Jyotsna W, Kaur G (2014). Comparison of posterior and subcostal approaches to ultrasound-guided transverse abdominis plane block for postoperative analgesia in laparoscopic cholecystectomy. J Clin Anesth.

[17] Shin HJ, Oh AY, Baik JS, Kim JH, Han SH, Hwang JW (2014). Ultrasound-guided oblique subcostal transversus abdominis plane block for analgesia after laparoscopic cholecystectomy: a randomized, controlled, observer-blinded study. Minerva Anestesiol.

[18] Suseela I, Anandan K, Aravind A, Kaniyil S (2018). Comparison of ultrasound-guided bilateral subcostal transversus abdominis plane block and port-site infiltration with bupivacaine in laparoscopic cholecystectomy. Indian J Anaesth.

[19] Tolchard S, Davies R, Martindale S (2012). Efficacy of the subcostal transversus abdominis plane block in laparoscopic cholecystectomy: comparison with conventional port-site infiltration. J Anaesthesiol Clin Pharmacol.

[20] Christopher Blom S, Kai Henrik Wiborg L, Christian R, Jakob K, Claus Anders B. Cutaneous sensory block area of the ultrasound-guided subcostal transversus abdominis plane block: an observational study. Regional Anesthesia &amp; Pain Medicine. 2023:rapm-2023-104753. doi: 10.1136/rapm-2023-104753 10.1136/rapm-2023-104753 37640451

[21] Kwon HJ, Kim YJ, Kim Y, Kim S, Cho H, Lee JH, Kim DH, Jeong SM (2023). Complications and technical consideration of Ultrasound-guided Rectus Sheath blocks: a retrospective analysis of 4033 patients. Anesth Analg.

[22] Rahiri J, Tuhoe J, Svirskis D, Lightfoot NJ, Lirk PB, Hill AG (2017). Systematic review of the systemic concentrations of local anaesthetic after transversus abdominis plane block and rectus sheath block. Br J Anaesth.

[23] Knudsen K, Beckman Suurkula M, Blomberg S, Sjovall J, Edvardsson N (1997). Central nervous and cardiovascular effects of i.v. infusions of ropivacaine, bupivacaine and placebo in volunteers. Br J Anaesth.

[24] Scott DB, Lee A, Fagan D, Bowler GM, Bloomfield P, Lundh R (1989). Acute toxicity of ropivacaine compared with that of bupivacaine. Anesth Analg.

[25] Sun Q, Liu S, Wu H, Ma H, Liu W, Fang M, Liu K, Pan Z (2019). Dexmedetomidine as an adjuvant to local anesthetics in Transversus Abdominis Plane Block: a systematic review and Meta-analysis. Clin J Pain.

[26] Lacassie HJ, Rolle A, Cortínez LI, Solari S, Corvetto MA, Altermatt FR (2018). Pharmacokinetics of levobupivacaine with epinephrine in transversus abdominis plane block for postoperative analgesia after caesarean section. Br J Anaesth.

[27] Parker RK, Holtmann B, White PF (1992). Effects of a nighttime opioid infusion with PCA therapy on patient comfort and analgesic requirements after abdominal hysterectomy. Anesthesiology.

[28] Dolan R, Huh J, Tiwari N, Sproat T, Camilleri-Brennan J (2016). A prospective analysis of sleep deprivation and disturbance in surgical patients. Ann Med Surg (Lond).

[29] Myles PS, Weitkamp B, Jones K, Melick J, Hensen S (2000). Validity and reliability of a postoperative quality of recovery score: the QoR-40. Br J Anaesth.

